# The cervical microbiome of ewe breeds with known divergent fertility following artificial insemination with frozen-thawed semen

**DOI:** 10.1038/s41598-025-97735-4

**Published:** 2025-04-26

**Authors:** Simen Foyn Nørstebø, Sabrina Rodriguez-Campos, Özgün C. O. Umu, Laura Abril-Parreño, Marianne Dalland, Gregor Duncan Gilfillan, Sean Fair, Anette Krogenaes

**Affiliations:** 1 Bacteriology and Mycology Unit, Department of Paraclinical Sciences, Faculty of Veterinary Medicine, Norwegian University of Life Sciences, Elizabeth Stephansens vei 15, 1433 Ås, Norway; 2 Physiology of Reproduction Group, Department of Physiology, Faculty of Veterinary Medicine, International Excellence Campus for Higher Education and Research (Campus Mare Nostrum), University of Murcia, Murcia, Spain; 3 Institute for Biomedical Research of Murcia, IMIB-Pascual Parrilla, Murcia, Spain; 4 Department of Medical Genetics, Oslo University Hospital and University of Oslo, Oslo, Norway; 5 Laboratory of Animal Reproduction, Department of Biological Sciences, Biomaterials Research Cluster, Faculty of Science and Engineering, Bernal Institute, University of Limerick, Limerick, Ireland; 6 Reproduction Unit, Faculty of Veterinary Medicine, Norwegian University of Life Sciences, Ås, Norway

**Keywords:** Cervix, Microbiome, Pregnancy, Reproduction, Sheep, Reproductive disorders, Microbiome

## Abstract

The use of artificial insemination (AI) with frozen-thawed semen in sheep is limited internationally due to low pregnancy rates. An exception is Norway, where high success rates routinely occur following vaginal deposition of frozen-thawed semen during natural estrus. Previous research suggests that breed-specific differences in pregnancy rates may result from impaired cervical sperm transport. This study compared cervical microbiomes among sheep breeds with known differences in pregnancy rates after AI. Cervical samples were collected from Suffolk (low fertility) and Belclare (medium fertility) breeds in Ireland, and Norwegian white sheep (NWS) and Fur breeds (both high fertility) in Norway, during the follicular phase of both natural and synchronized estruses, and the luteal phase of synchronized estrus. Amplicon sequencing revealed significantly higher bacterial abundance during the follicular phase in the low-fertility Suffolk breed compared to high-fertility breeds. Alpha diversity was higher in Suffolk and Belclare breeds, especially during the natural follicular phase, coinciding with pronounced beta diversity differences among breeds. Genus *Histophilus* was the top feature leading to microbial differences between ewe breeds and types of cycle. Ewe breed was the main driver of cervical microbial composition; increased microbial load in lower-fertility breeds may negatively impact sperm survival/transport, hampering AI success.

## Introduction

Artificial insemination (AI) with frozen-thawed semen is the single most important technique for the genetic improvement of livestock as elite males can be used extensively across geographical regions while also facilitating infection control. However, in sheep, the use of AI with frozen-thawed semen is hampered internationally by low pregnancy rates (< 30%)^1^, limiting its widespread use. The exception to this is in Norway where vaginal (shot-in-the-dark) insemination of frozen-thawed semen during natural estrous cycles routinely yield pregnancy rates of more than 60% at the farm level for ewe breeds such as Norwegian White Sheep (NWS) and Norwegian Fur Sheep (Fur)^2^. Previous studies have shown that differences in pregnancy rates following AI are not due to differences in semen quality but are related to the ewe breed used^3^. These ewe breed differences cannot be explained by oocyte quality^4^ or hormonal profiles in the periovulatory period^5^. Interestingly, there were significant differences in fertilization rates between the Suffolk (low fertility breed) and Belclare (medium fertility breed) following cervical AI with frozen-thawed semen, but not after laparoscopic insemination^6^, indicating that the ability of sperm to traverse the cervix differs between ewe breeds. Our group conducted several studies with the aim of comparing cervical function in European ewe breeds (across Norway, France and Ireland) that are known to have different pregnancy rates following cervical AI with frozen-thawed semen. A comparison of the cervical gross anatomy and mucus properties between these ewe breeds could not establish a relationship between these parameters and pregnancy rates^7^. A transcriptomic analysis of post-mortem cervical tissue samples identified differential expression of genes involved in the immune response at the follicular phase of a natural cycle^8^. Higher levels of pro-inflammatory cytokines were also found in the low fertility Suffolk breed across the follicular^8,9^ and luteal^10^ phases of a synchronized cycle indicating a heightened cervical immune response in the low fertility breed (Suffolk). In line with this, we have recently characterized the metabolome of the cervical secretions in the same ewe breeds. Biochemicals produced by mixed anaerobic bacteria such as 3-indoxyl sulfate, cadaverine and putrescine were identified in higher levels in the low fertility Suffolk breed indicating that the Suffolk breed may have a higher cervical bacterial load compared to higher fertility ewe breeds of NWS and Fur^11^.

Increasing evidence suggests that the female upper reproductive tract harbors a distinct microbiome, challenging the traditional notion that it is sterile^12^. Previous studies have shown correlations between the microbiome of both the lower and upper parts of the female reproductive tract and pregnancy outcomes across various animal species^12,13^. For instance, specific microbiomes have been described in the endometrium of healthy cows and cows with metritis^14,15^. In the case of sheep, studies indicate that the vaginal microbiome is markedly diverse, dominated by taxa such as Proteobacteria, Fusobacteria, Bacteroidetes, Firmicutes, and Actinobacteria, leading to a higher pH range of 7.0 to 7.2^16–18^. To our knowledge, the microbiome of the cervix in ewes remains poorly characterized. We hypothesized that ewes harbor a cervical microbiome that may influence sperm transport through the cervix and that compositional differences between ewe breeds contribute to variations in reproductive outcomes. Additionally, we reasoned that the cervical microbiome undergoes changes throughout the estrous cycle in response to physical and hormonal shifts and that the mid-cycle microbiome may play a role in priming the reproductive tract for the subsequent cycle. The aim of this study was to investigate the cervical microbiome of ewe breeds with known differences in fertility following AI with frozen-thawed semen. Specifically, we characterized and compared the cervical microbiome of ewe breeds known to have high (NWS and Fur), medium (Belclare) and low (Suffolk) pregnancy rates following AI with frozen-thawed semen. Cervical samples were collected at the follicular and luteal phases of the estrous cycle. For the follicular phase, we included samples obtained after both natural and synchronized estrous cycles, as Norwegian ewes are routinely inseminated during a natural estrous cycle by vaginal deposition of semen, unlike the synchronized estrous cycle and cervical deposition commonly employed in other countries.

## Results

### Lower fertility ewe breeds have a higher cervical bacterial abundance

Total bacterial counts were calculated by quantitative PCR analysis using universal primers for the 16S rRNA gene^19^ (Supplementary Fig. 1). Samples were taken at the follicular phase of a natural estrous cycle and at the follicular and luteal phases of a synchronized cycle and showed median values of 3.77 (IQR 3.14–4.47), 3.36 (IQR 2.61–4.17) and 2.87 (IQR 2.23–3.70) log_10_ 16S copies per sample, respectively. In comparison, the median value of log10 16S rRNA gene copies in the blank extraction controls (unused swabs) was 2.41 (IQR 1.93–2.72). There were no differences between phases of the cycle or between synchronized and natural estrous cycles, but there was a phase by breed interaction on relative abundance (*P* < 0.05). At the follicular phase of a natural estrous cycle, the bacterial abundance levels were significantly higher in Suffolk (low fertility) and Belclare (medium fertility) ewes compared to NWS and Fur (both high fertility) ewes (Belclare vs. Fur: *p* < 0.001; Belclare vs. NWS: *p* < 0.001; Suffolk vs. Fur: *p* < 0.02; Suffolk vs. NWS: *p* < 0.01). Similarly, at the follicular phase of a synchronized cycle, the low fertility Suffolk had higher bacterial abundance compared to the Fur (high fertility) (Supplementary Fig. 1). In the luteal phase of the synchronized cycle, there were no significant differences between ewe breeds in bacterial abundance.

### Characterization of bacterial microbiome by 16S sequencing after removal of contaminants

To investigate the cervical microbiome of the four ewe breeds, cervical swabs were subjected to sequencing, quality-controlled, and clustered into amplicon sequence variants (ASVs). In total, 11.92 million paired-end raw reads were obtained from the 110 samples, and 7.45 million non-chimeric reads remained after filtering (average per sample: 67,743 reads, max: 197,659 reads, min: 20,087 reads), representative of 8232 ASVs (Supplementary Table 1).

**Fig. 1:**
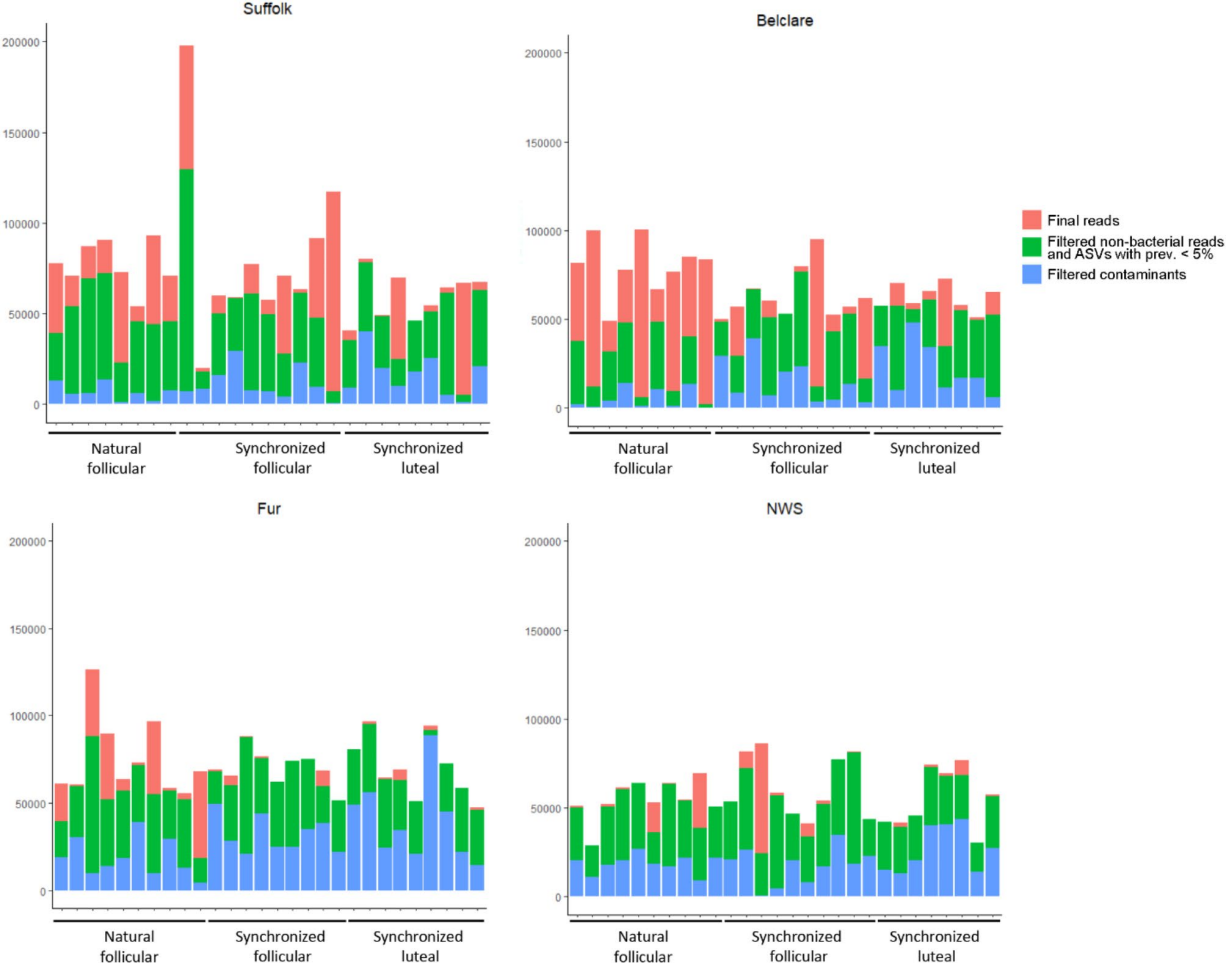
Bar plots showing reads classified as identified contaminant amplicon sequence variants (ASVs); ASVs filtered due to identification as host reads, Archaea, chloroplasts, or mitochondria, or due to presence in less than 5% of the total number of samples; and ASVs kept in the final data set for cervical samples from Suffolk, Belclare, Fur and Norwegian white sheep (NWS).

Due to the low microbial load in our samples, blank extraction controls and a mock community dilution series were used in conjunction with the R package decontam to identify contaminants present in the dataset as described by Karstens and co-workers^20^. Analysis of the control samples identified 392 ASVs, including the expected eight mock ASVs and one ASV that differed from the expected mock sequence by one nucleotide (Supplementary Fig. 2). By comparing the prevalence of ASVs in control samples to the sheep samples, 71 ASVs were identified as contaminants. After an additional manual check, an ASV affiliated with *Undibacterium* (ASV2) was also identified as a contaminant due to the high abundance in all negative controls. Overall, 72 ASVs were detected as contaminants and filtered out (Fig. 1 and Supplementary Table 2). To corroborate the filtering procedure, filtered reads were correlated to qPCR results indicative of bacterial abundance in the cytobrush samples.

### Composition and abundance

**Fig. 2:**
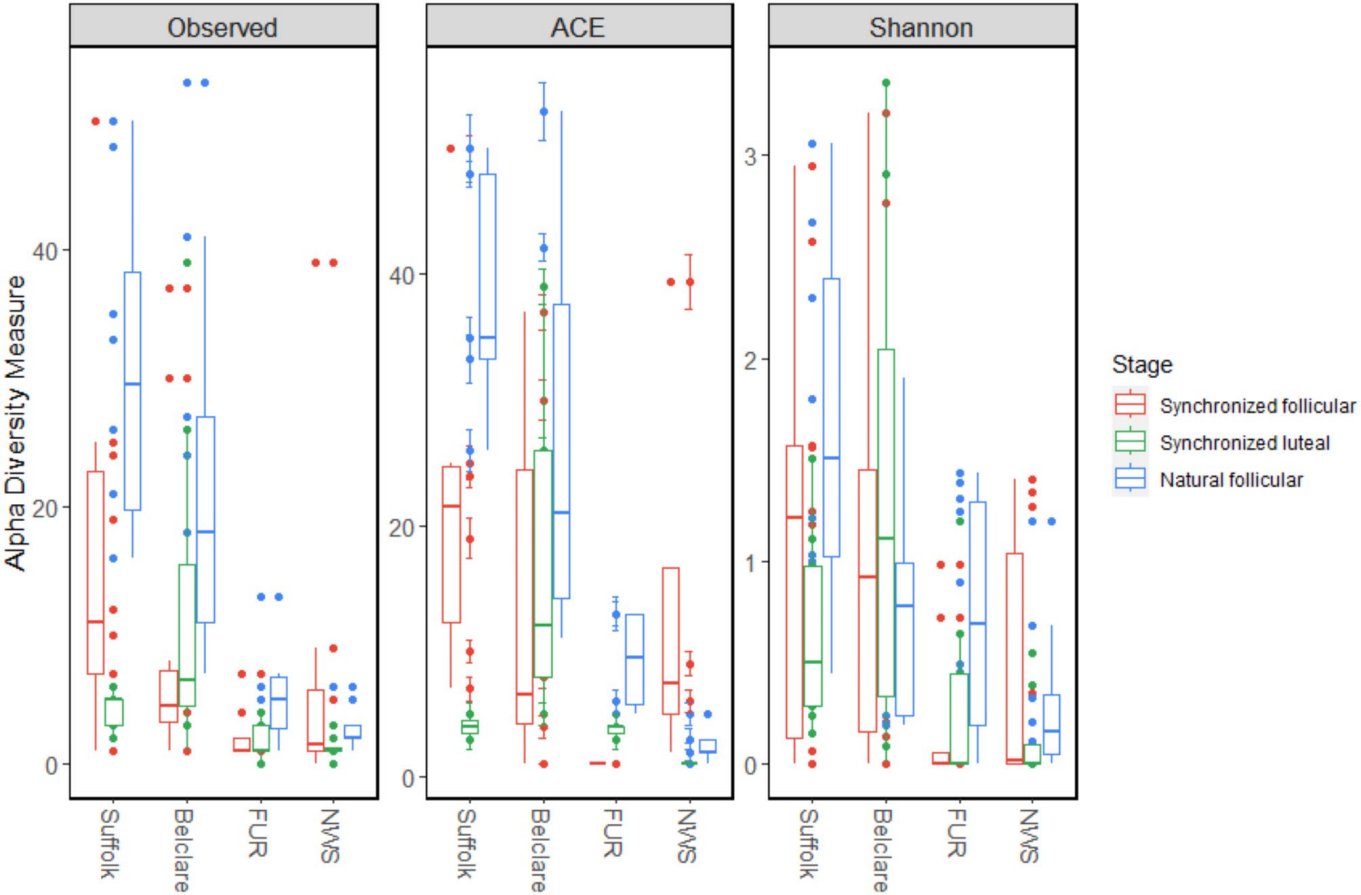
Box plots illustrating the alpha diversity of the cervical microbiome estimated by observed features (Observed), abundance-based coverage estimator (ACE) and Shannon diversity index in Suffolk (*n* = 27), Belclare (*n* = 27), Fur (*n* = 28) and Norwegian White Sheep (NWS; *n* = 28) at the follicular phase of a natural estrus, and at both the follicular and luteal phases of a hormonally synchronized estrus. No statistically significant differences in alpha diversity were observed when comparing phases of the estrous cycle within each breed. All p-values are shown in Supplementary Table 5.

**Fig. 3:**
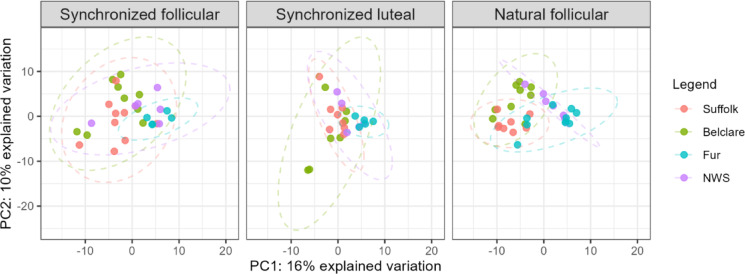
Unsupervised principal component analysis (PCA) of the cervical bacterial microbiome in Suffolk, Belclare, Fur and Norwegian White Sheep (NWS) at the follicular phase of a natural estrus (Suffolk: *n* = 8, Belclare: *n* = 9, Fur: *n* = 10, NWS: *n* = 6) and follicular (Suffolk: *n* = 10, Belclare: *n* = 9, Fur: *n* = 5, NWS: *n* = 6) and luteal phases of a hormonally synchronized estrus (Suffolk: *n* = 8, Belclare: *n* = 7, Fur: *n* = 5, NWS: *n* = 5).

The alpha diversity (microbial diversity within a sample) and beta diversity (similarity between groups) were assessed for the four ewe breeds at the follicular phases of both a natural and synchronized estrous cycle, and at the luteal phase of a synchronized estrous cycle. When assessing phases of the estrous cycle within each breed, no statistically significant differences in alpha diversity were observed between estrous phases or between a natural and synchronized cycle (Fig. 2, Supplementary Table 5). A comparison between ewe breeds showed a significantly higher alpha diversity in Suffolk and Belclare compared to both Fur and NWS for the three estimators: (1) observed features, (2) abundance-based coverage estimator (ACE) and (3) Shannon diversity index (Table 1). When comparing breeds by assessing each phase of the estrous cycle separately, the largest contribution to differences between breeds was observed in the follicular phase of a natural cycle (Fig. 2; observed features: Suffolk vs. NWS: *p* = 0.025, Suffolk vs. Fur: *p* = 0.026, Belclare vs. NWS: *p* = 0.017, Belclare vs. Fur: *p* = 0.13). We also found that, within each breed, there was no consistent pattern in alpha diversity results between different phases (Fig. 2, Supplementary Table 5). The beta diversity, as visualized by a PCA, was lower at the synchronized luteal phase compared to the synchronized follicular phase within the Belclare and Suffolk breeds (Fig. 3). A higher diversity was observed at the follicular phase of a synchronized estrus than at a natural estrus in Suffolk, but not in the other breeds. However, separation of breeds was observed both at the follicular phase of a natural and a synchronized estrus. To further investigate the differences between breeds in the follicular phase, we performed a supervised multivariate analysis (sPLS-DA), which confirmed that the beta diversity of Suffolk and Belclare was higher than both Fur and NWS (Supplementary Fig. 3). Visual comparison by sPLS-DA of the different breeds sorted by the follicular phase of either a natural or a synchronized estrus, also showed that, for Suffolk and NWS, there was a higher beta diversity at the synchronized follicular phase than at a natural follicular phase, while an opposite pattern was observed for Fur (Supplementary Fig. 4). Furthermore, Suffolk ewes sampled at the synchronized and natural follicular phases did not overlap, in contrast to the other breeds. No p-values were reported because this was an exploratory data analysis, but 95% confidence ellipses that highlight the strength of discrimination were included in the plots.

**Table 1:** Pairwise comparison between alpha diversity indices observed features, abundance-based coverage estimator (ACE) and Shannon in all cervical samples from Belclare, Suffolk, Fur and Norwegian white sheep (NWS; *n* = 27 to 28 per breed) ewes. The comparisons include samples taken at the follicular phase and luteal phase of a synchronized estrus, as well as the follicular phase of a natural estrus. Data were analyzed using the Wilcoxon rank sum test with continuity correction and the Holm correction as the p-value adjustment method (significant p-values in bold).

Breed groups	*P*-value		
	Observed features	ACE	Shannon
Suffolk vs. Belclare	0.980	0.804	0.995
Suffolk vs. NWS	**< 0.001**	**0.006**	**< 0.001**
Suffolk vs. Fur	**< 0.001**	**0.016**	**0.004**
Belclare vs. NWS	**< 0.001**	**0.016**	**0.002**
Belclare vs. FUR	**< 0.001**	**0.033**	**0.011**
NWS vs. FUR	0.980	0.804	0.995

**Fig. 4:**
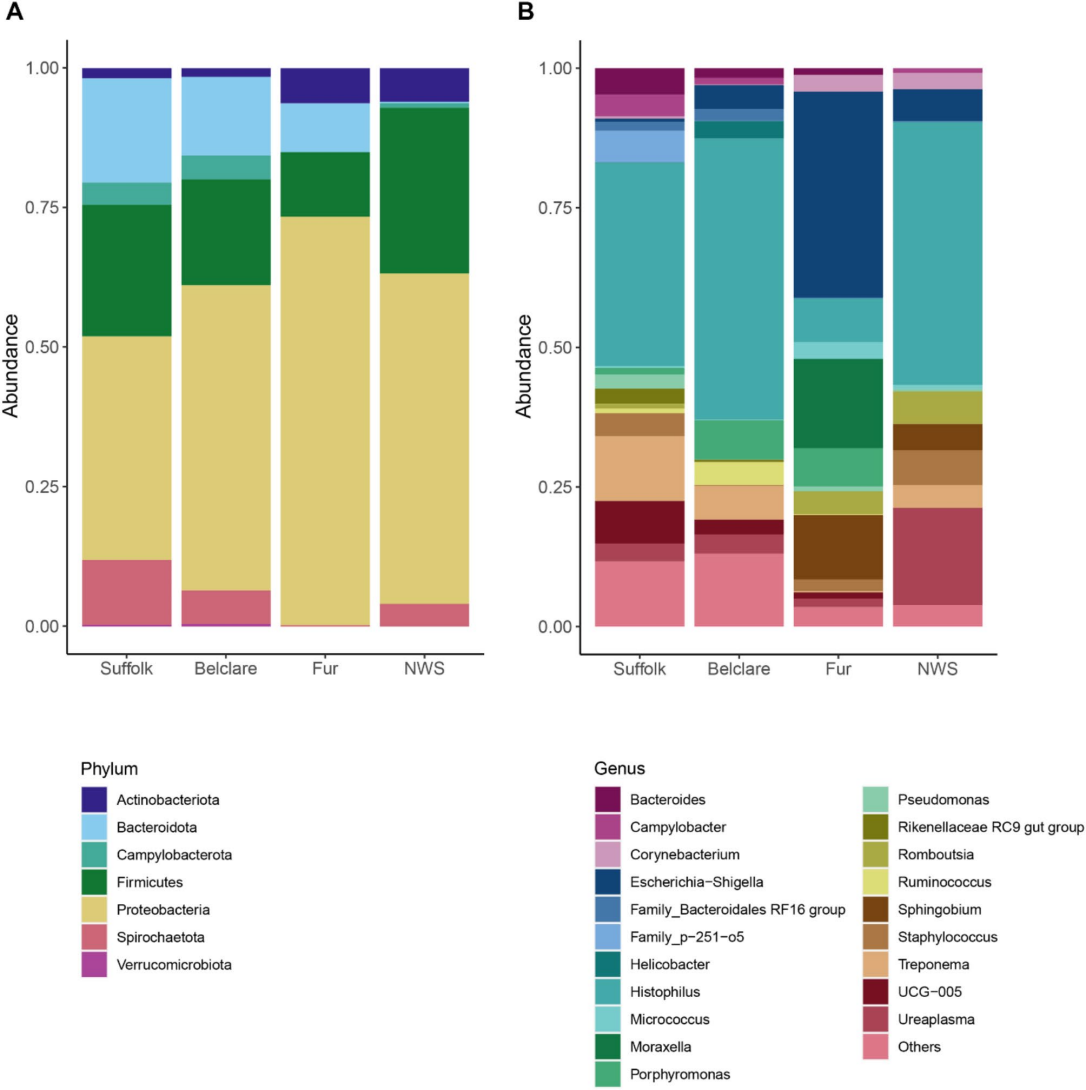
Taxa bar plots showing the cervical microbiome composition for Suffolk, Belclare, Fur, and Norwegian White Sheep (NWS) at the phylum (**A**) and genus (**B**) levels. Data are combined within breed for the follicular phase of a natural and synchronized estrous, as well as at the luteal phase of a synchronized cycle. Only the top 20 genera are shown.

The most abundant phyla across all breeds (for all phases and types of cycle) were Proteobacteria (Suffolk: 40.1%, Belclare: 54.7%, Fur: 73.2%, NWS: 59.2%), Firmicutes (Suffolk: 23.6%, Belclare: 18.9%, Fur: 11.5%, NWS: 29.7%), Bacteroidota (Suffolk: 18.7%, Belclare: 14.1%, Fur: 8.8%, NWS: 0.3%), and Spirochaetota (Suffolk: 11.6%, Belclare: 6.0%, Fur: 0.2%, NWS: 4.1%) (Fig. 4A). The abundance of Campylobacterota, represented by two ASVs, was higher in Suffolk and Belclare (4.0 and 4.3%, respectively) than in Fur and NWS (0% and 0.8%). Conversely, Actinobacteriota abundance was higher in Norwegian breeds (Fur: 6.3%, NWS: 6.0%) than in Suffolk (1.8%) and Belclare (1.5%). The top 20 most abundant genera in each breed, accounting for 92.0% of all reads, are shown in (Fig. 4B). Among the top 20, 13 taxa at genus level were shared across all ewe breeds. None of the taxa were unique to any of the breeds.

**Fig. 5:**
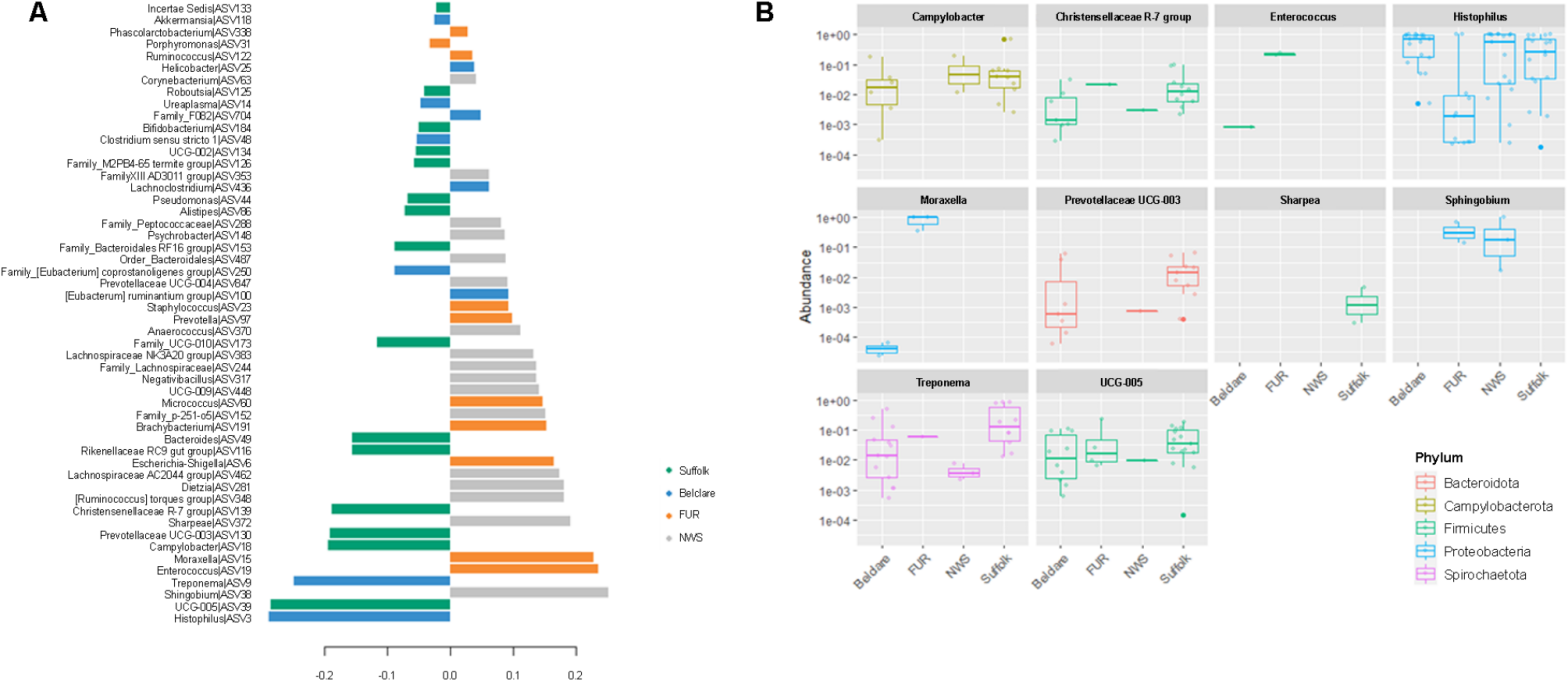
Linear discriminant analysis effect size (LEfSe)-like plot generated using mixOmics (**A**). The plot illustrates the contribution to the first component of the supervised partial least-squares discriminant analysis (sPLS-DA) for ewes sampled at the follicular phase of either natural or synchronized estrus, as presented in (Supplementary Fig. 3).The bars represent amplicon sequence variants (ASVs) that were found more abundant in one of the ewe breeds [Suffolk, green; Belclare, blue; Fur, orange; Norwegian White Sheep (NWS), gray] based on effect size (LDA score, x-axis) and contribute significantly to their separation. (**B**) Box plots showing abundance levels in Suffolk, Belclare, Fur, and NWS of the top 10 ASVs contributing to the sPLS-DA model.

Figure 5, based on the sPLS-DA model presented in (Supplementary Fig. 3), highlights the ASVs identified as the most important contributors to the separation of breeds during the follicular phase, where the greatest differentiation between breeds were observed. Out of the ten ASVs that contribute the most to the separation of breeds, ASV3 (genus *Histophilus*, phylum Proteobacteria) was the main contributor and was present at highest relative abundance in Belclare (50.3%) and NWS (47.1%), followed by Suffolk (36.4%) and Fur ewes (7.8%). The second most important contributor to the separation of breeds was *Ruminococcaceae* UCG-005 (ASV39), which was present at the highest relative abundance in Suffolk (7.6%), followed by Belclare (2.7%), Fur (1.1%), and NWS (0.03%). The third most important contributor was *Sphingobium* (ASV38), which was only present in Fur and NWS at 11.6% and 4.7% relative abundance, respectively. The *Treponema* ASV9 was present across all ewe breeds, but was most abundant in Suffolk (11.6%), followed by Belclare (6.0%), NWS (4.1%), and Fur sheep (0.2%). *Enterococcus* and *Moraxella* were found among the top 20 genera in Fur sheep, with a relative abundance of 2.2% and 16.0% of the corresponding ASV19 and ASV15 (Fig. 5), respectively. Neither ASV19 nor ASV15 were found in NWS and were almost absent (relative abundance < 0.0001%) from Suffolk and Belclare. *Campylobacter* (ASV18) was most abundant in Suffolk (3.9%), followed by Belclare (1.1%) and NWS (0.8%), but was not present in Fur. The *Prevotellaceae* UCG-003 (ASV130) was only present in Suffolk and Belclare at a relative abundance of 0.8% and 1.1%, respectively. Similarly, *Sharpea* (ASV372) was not present in Fur sheep and NWS but was found at a relative abundance of 0.3% in Belclare and 0.05% in Suffolk. The tenth most important contributor was *Christensenellaceae* R-7 group (ASV372), which was most abundant in Suffolk (1.2%), followed by low abundances in Belclare (0.2%), NWS (0.08%), and Fur (0.01%).

When analyzing these top ten contributors by follicular phase of either natural or synchronized estrus (Supplementary Fig. 5), seven of the contributors could be attributed to differences in the natural cycle (*Histophilus* ASV3, *Ruminococcaceae* UCG-005, *Treponema* ASV9, *Enterococcus* ASV19, *Moraxella* ASV15, *Campylobacter* ASV18, and *Prevotella* ASV97) while the other three could be attributed to differences in the synchronized cycle (*Sphingobium* ASV38, *Sharpea* ASV372, and *Christensella* ASV139).

## Discussion

To the best of our knowledge, this is the first published report of the microbiota composition of the sheep cervical mucosa. In this study, we used a novel sheep model to investigate the cervical microbiome across the estrous cycle of healthy ewes of four breeds known to have different pregnancy rates, likely due to divergent cervical sperm transport. We also assessed the effect of estrus synchronization on the cervical microbiome. The main findings were that ewe breed was the main driver of the observed differences in the cervical microbial composition, and that the differences between ewe breeds were most highly pronounced in the follicular phase of a natural estrus.

### Ewe breed as the main driver of cervical microbial diversity

The bacterial abundance levels, as measured by 16S qPCR, were significantly higher in Suffolk (low fertility) and Belclare (medium fertility) compared to NWS and Fur (both high fertility) ewe breeds at the follicular phase of the natural cycle. The low and medium fertility breeds were reared in Ireland, whereas the high fertility breeds were reared in Norway. This could have influenced the bacterial differences found in this study. Regardless, our findings agree with our previously published RNA-sequencing studies on these same ewe breeds, which demonstrated that low fertility ewe breeds have a heightened constitutive cervical immune response. Furthermore, Suffolk ewes had higher bacterial abundance than the Fur ewes at the follicular phase of the synchronized cycle. We also found that the two ewe breeds with the lowest fertility (Suffolk and Belclare) demonstrated higher alpha diversity than the two breeds with high fertility (NWS and Fur), and these differences were found mainly at the follicular phase of the natural cycle. In sheep, contradictory results have been reported regarding the association between alpha diversity of the vaginal microbiome and reproductive rates. Koester and colleagues^21^ did not find differences in alpha diversity of the vaginal microbiome in two related ewe breeds (Hampshire and Hampshire cross Suffolk), but differences between pregnant and non-pregnant ewes were reported. A possible explanation for the complexity of this issue could be the effect of the gut microbiota, which in humans has been shown to impact the reproductive endocrine system and fertility^22^. Other studies on dairy cattle breeds found no differences in alpha diversity between pregnant and non-pregnant cows or between herds with differing reproductive rates^15^.

### Phase and type of estrous cycle impacts microbial diversity

From both physiological and clinical perspectives, a less diverse microbiome is expected during the luteal phase than the follicular phase. Additionally, hormone levels in the cervix are likely to influence the bacterial community throughout the cycle. Due to high variance within groups, we did not identify significant differences in alpha diversity among estrous phases. However, the highest variation in alpha diversity indices between phases of the cycle was observed in the low fertility Suffolk breed, indicating that the other breeds harbored a more robust microbiota across the phases of the estrous cycle. While we observed a consistent trend of higher bacterial abundance levels at the follicular phase compared to the luteal phase, the alpha diversity estimators did not follow the same pattern. In Belclare and Fur ewes, the alpha diversity was higher at the luteal phase than at the follicular phase of a synchronized cycle, while the opposite was observed in Suffolk and NWS. In healthy cattle, the alpha diversity of the vagina was found to be significantly lower at the luteal phase compared to the follicular phase^23^, implying that progesterone reduces bacterial diversity. In healthy women, a decrease in species richness and evenness in the uterus was shown during the luteal phase, while the opposite was observed in women with recurrent miscarriage and implantation failure^24^. The difference between NWS and Suffolk ewes (with different pregnancy rates) is not explained by alpha diversity alone, but when looking at bacterial abundance levels and alpha diversity in combination, it appears that Suffolk ewes are more likely to experience disturbances in their microbiota. This is also reflected in the high beta diversity of the Suffolk ewes compared to the other ewe breeds, as shown in the PCA plot (Fig. 3) and sPLS-DA plots (Supplementary Fig. 3). One could discuss whether a shift in bacterial community could also influence mucus pH, and further sperm survival and transport. In a previous study by Richardson and colleagues^25^, no effect of breed or time after sponge removal on mucus pH was observed, and therefore pH in mucus was not measured in the present study. To avoid geography as a possible confounding factor, the experiment would ideally have to be carried out at the same location, which was logistically impossible in this study. Thus, it is important to be aware of potential geographical variations in the microbiome. Notably, in a global comparison of the rumen microbial community, Henderson and colleagues^26^ concluded that a core microbiome exists regardless of geographical location. In contrast, Wege Dias and colleagues^27^ summarized that the bovine vagina does not appear to have a core microbiome but is instead shaped more by core metabolic pathways. This aspect would require further study in an extended experimental setting including more animals and functional analysis.

### Taxonomic findings

Across all four ewe breeds, the cervical microbiome was dominated by Proteobacteria, Firmicutes, Bacteroidota and Spirochaetota. This is in line with previous studies on the vaginal microbiome of ewes^16–18,21^. While Proteobacteria and Firmicutes were found at similar abundance levels in all ewe breeds, the abundances of Bacteroidota and Campylobacterota were higher in Suffolk and Belclare compared to NWS and Fur. Again, some of these differences may reflect variations in bacterial communities influenced by geographical factors, which could contribute to the observed breed differences. For instance, the spirochetes *Treponema* are involved in contagious ovine digital dermatitis that is widespread in Ireland but has not been described in Norway^28,29^. Interestingly, some of the ASVs contributing to the separation of breeds in the sPLS-DA plot and belonging to these two phyla have previously been associated with reproductive disorders and impaired spermatozoa function, as described in the following. In our study, *Campylobacter* spp. were more abundant in Suffolk and Belclare, while they were found in lower amounts in NWS and were absent in Fur. *Campylobacter* spp. are found in the intestinal and genital tracts of ruminants with a wide geographic distribution. In a study of the bovine vaginal microbiota, *Campylobacter* spp. were exclusively associated with cows with reproductive disorders^30^. Furthermore, a highly pathogenic strain of *C. jejuni* has gained importance in recent years as the predominant abortive agent in sheep in North America^31^. *C. fetus* subsp. *fetus* has been shown to have a detrimental influence on in vitro sperm quality in rams^32^. It would be interesting to further investigate the effect of the microbiome post AI to assess whether certain taxa have detrimental or beneficial effects on sperm survival and/or transport.

The *Bacteroides* genus was also found in higher levels in the low fertility Suffolk breed compared to the other ewe breeds. Bacteria of this genus, e.g. *Bacteroides fragilis* or *Bacteroides thetaiotamicron*^33^, produce enzymes called sialidases that cleave the sialic acid termini on the *O*-glycans, also called sialidases. This allows access to the underlying sugars of the glycan chains^34^ and thus in certain conditions, such as post-antibiotic treatment, the increased levels of free sialic acid have been shown to promote the expansion of pathogens such as *Clostridium difficile*, *Salmonella* sp., and *Escherichia coli*^35^. In agreement with this, we have previously reported^36^, using the same ewes as the current study, that the low fertility Suffolk breed had the highest levels of free sialic acid, specifically the acetylated (Neu5,9Ac2) sialic acid, which has been reported to be more susceptible to the action of the sialidases from Bacteroides^37^. Therefore, the cervix of the Suffolk seems to have a suboptimal environment, which can lead to perturbations in the microbiota and potentially reduced cervical sperm survival and/or transport. A sub-optimal and pro-inflammatory environment that could have a negative effect on cervical sperm transport was previously reported in the Suffolk breed, where reduced levels of lipids involved in the resolution of inflammation were identified in comparison to high fertility ewe breeds^38^.

Although *Proteobacteria* had similar abundance levels across breeds, the highest contribution to the sPLS-DA plot was observed for an ASV identified as *Histophilus*, belonging to this phylum, which also accounted for an average of 58.7% of all ASVs and a sample average of 35.4%. In a comparison of vaginal microbiomes of ewes, *Histophilus* was found to be overrepresented in the ewes that failed to establish pregnancy^16^. In a previous study in cattle, *Histophilus* was among the top three dominant taxa in the vaginal microbiome of unhealthy cows^39^. It was increased in vaginal samples from heifers that did not establish a pregnancy^40^. In contrast to this, we found that *Histophilus* was present in lower levels in the Fur (high fertility breed), but in higher levels in the high fertility NWS breed compared to Suffolk, suggesting that *Histophilus* does not explain the differences in cervical sperm survival and/or transport between these ewe breeds.

### Methodological considerations

The cervix acts as a major barrier to pathogens and infection and is expected to have a low microbial load. An important issue in microbiome studies is the presence of exogenous DNA that can arise from sequencer errors or laboratory contaminants. We included several measures to limit effects of contaminants that could have been introduced during sample preparation, DNA extraction, and sequencing. Our initial investigation confirms that in low microbial biomass samples, exogenous DNA may have an impact on the data interpretation^41^. Low biomass samples have been defined as biological samples that contain similar quantities of target microbial DNA in the sample compared to negative controls^42^. Different types of control samples, as well as computational approaches, have been proposed in recent years to address this problem^41,43^, but no standard approach is available to date. To address the possible problems arising from processing low biomass samples like cervical swabs, we followed the RIDE checklist for recommended experimental criteria^41^, except for the inclusion of sampling blanks due to the retrospective character of the study. We also included a mock community dilution series as a control to establish a filtering cut-off as recommended by Karstens and colleagues^20^. Additionally, known reagent contaminants were removed manually.

## Conclusions

This is the first published study describing the cervical microbiome of healthy sheep and how it can be influenced by estrus synchronization, phase of the estrous cycle, and ewe breed. We found that ewe breed was the main driver of the cervical microbial composition, with bacterial abundance levels being significantly higher in the ewe breeds with impaired cervical sperm survival and/or transport. The same breeds also had higher alpha diversity than the breeds with higher pregnancy rates, and this was most pronounced in the follicular phase of a natural cycle. The greatest separation in beta diversity between breeds was also seen at the follicular phase of a natural cycle. The low fertility breed Suffolk showed the highest variation in alpha diversity indices between phases of the estrous cycle, indicating a less robust cervical microbiota. Further work is now required to study the mechanisms by which specific microbes can affect sperm survival and/or transport in vivo.

## Methods

### Description of experimental animals and sampling

**Fig. 6:**
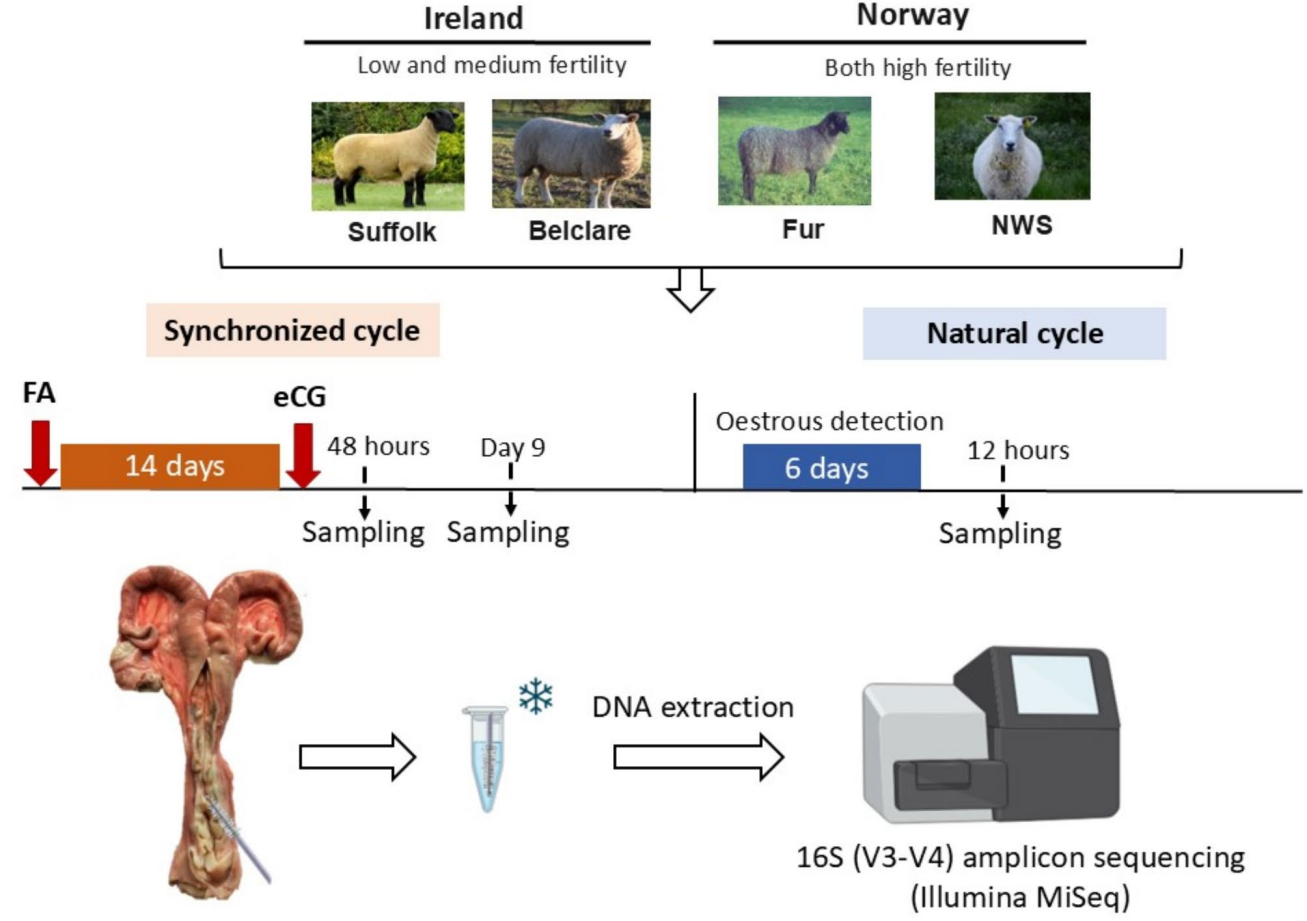
Timeline of experimental model for sample collection. All ewes (*n* = 27 to 28 per ewe breed) were synchronized using intravaginal progestogen sponges [Chronogest 20 mg flugestone acetate (FA)] for 14 days, followed by intramuscular administration of 400 IU equine chorionic gonadotropin (eCG) at sponge removal. Reproductive tracts were recovered 48 h later (synchronized follicular) and 9 days later (synchronized luteal). The remaining ewes returned to heat naturally, detected twice daily with a ram fitted with an apron, and slaughtered 12 h after heat detection (natural follicular). Once the reproductive tracts were recovered, the cervix was opened longitudinally and a cytobrush was placed in the mid-region where it was rotated clockwise to obtain cellular material.

The animal model was previously described as part of a larger study on ewe breed effects on mucus properties and anatomical characteristics across the estrous cycle at both a synchronized and a natural estrus (Fig. 6)^7^. Commercially bred purebred ewes were used, and all experimentation was carried out on licensed research farms under the control of research institutes in Ireland (Teagasc Research Centre, Athenry) and Norway (Section for Small Ruminant Research and Herd Health, NMBU, Faculty of Veterinary Medicine, Sandnes). The Suffolk and Belclare ewes were born and remained in Ireland, whereas the NWS and Fur ewes were born and remained in Norway. Within each country, the breeds were managed under similar environmental and nutritional conditions. All ewes were maintained indoors for the duration of the experiment, with *ad libitum* access to forage and clean water. All the ewes used in this study were multiparous in a range of 4 to 5 years old. The experiment was carried out during one season (September to February) and included four European ewe breeds from two countries in the northern hemisphere: Suffolk (low fertility, *n* = 27) and Belclare (medium fertility, *n* = 27) from Ireland, as well as Norwegian white sheep (NWS; high fertility, *n* = 28) and Fur sheep (high fertility, *n* = 28) from Norway.

Briefly, synchronization of estrus was performed using intravaginal progestogen sponges (Chronogest 20 mg FGA, Intervet, Boxmeer, The Netherlands) for 14 days, and 400 IU of equine chorionic gonadotropin (Intervet) was administered intramuscularly at sponge removal. Ewes (*n* = 8 to 10 per ewe breed for each phase of the cycle) were either slaughtered 48 h later (Synchronized follicular) or 9 days later (Synchronized luteal) or were allowed to come back into heat naturally, assessed twice a day using a ram fitted with an apron, and slaughtered 12 h after heat detection (Natural follicular). Slaughter took place at approved slaughterhouses following the Regulation (EC) No. 853/2004 of the European Parliament and of the Council of 29 April 2004, laying down specific hygiene rules for food of animal origin (accessed on 5 August 2024, available online http://data.europa.eu/eli/reg/2004/853/oj). The reproductive tracts were recovered, and the cervix opened longitudinally. A cytobrush (Minitube, Grenoble, Auvergne Rhone Alpes, France) was placed in the mid-region, where it was rotated clockwise a full 360 degrees to obtain cellular material. Then, the cytobrush was carefully removed, detached from the rod mechanism, and placed in a 1.5 mL sterile Eppendorf containing RNAlater (Sigma-Aldrich, St. Louis, US). All samples were then transported to Norway for DNA extraction, quantification, and 16S RNA sequencing.

### DNA extraction

DNA was extracted using the IndiSpin Pathogen Kit (Indical Biosciences GmbH, Leipzig, Germany) with the following modifications: the tubes containing the cytobrushes were centrifuged at maximum speed in a tabletop centrifuge, and the resultant pellet was dissolved in 500 µl Buffer ATL and transferred to a Pathogen Lysis Tube S (Qiagen, Hilden, Germany) together with the cytobrush for mechanical lysis on a TissueLyser II (Qiagen) for 10 min at a frequency of 30 Hz. Finally, 400 µl of supernatant were used to continue the manufacturer’s protocol, doubling the amounts of required reagents; for elution, 75 µl of Buffer AVE was used.

With every batch of extractions (samples extracted over 11 batches with samples for each selected at random), a negative extraction control was included. The extractions were performed by three persons. To control for inter-person variation, this information was included in the metadata along with an assigned batch number. Additionally, to determine the appropriate filter level for downstream analysis as recommended for low biomass samples^20^, DNA extractions of eight rounds of a 3-fold serial dilution of ZymoBIOMICS™Microbial Community Standard (ZymoResearch, Irvine, CA, USA) were included, containing the following bacterial species: *Pseudomonas aeruginosa*, *Escherichia coli*, *Salmonella enterica*, *Lactobacillus fermentum*, *Enterococcus faecalis*, *Staphylococcus aureus*, *Listeria monocytogenes*, and *Bacillus subtilis*.

### Quantification of DNA

Quantification of the extracted DNA was performed using a Qubit 3.0 Fluorometer with the dsDNA Broad Range Assay Kit (Invitrogen, Eugene, OR, USA) and DNA purity was assessed using Nanodrop 1000 (Thermo Fisher Scientific, Waltham, MA, USA). Copies of the 16S rRNA gene per sample were determined by qPCR using a previously described primer set (forward primer: 5’-TCCTACGGGAGGCAGCAGT-3’; reverse primer: 5’-GGACTACCAGGGTATCTAATCCTGTT-3’)^19^ in a total volume of 20 µl per reaction on an Mx3005p Real-Time PCR System (Agilent Technologies, Santa Clara, CA, USA). The Master Mix for each reaction contained: 1X PowerUp™ SYBR™ Green Master Mix (Applied Biosystems™, Foster City, CA, USA), 0.2 µM of each primer, and 2 µl of template DNA. The cycling conditions were as follows: 2 min at 50 °C and 2 min at 95 °C, followed by 40 cycles of 15 s at 95 °C and 1 min at 60 °C, and dissociation for 1 min at 95 °C, 30 s at 55 °C, and 30 s at 95 °C. Bacterial abundance levels between treatments were compared using a Kruskal-Wallis rank sum test with post-hoc pairwise comparisons.

### 16S amplification and sequencing

Amplification of the 16S V3-V4 region was performed based on the procedure described in the Illumina application note^44^. The primer pair used in our study for amplification of the V3-V4 region of the 16S rRNA gene was previously evaluated by Klindworth and collaborators^45^, who concluded that it presented the best combination of overall coverage and phylum spectrum with the SILVA database^46^ that we used for taxonomic classification in this study. Briefly, cervical swab DNA concentrations were measured on a SpectraMax M3 fluorometric plate reader (Molecular Devices, San Jose, CA, USA) using Quant-IT BR Assay reagents (Thermo Fisher Scientific, Waltham, MA, USA) and diluted to 5 ng per µl. Cervical swab DNA (12.5 ng per sample), negative control (water), and positive control (ZymoBIOMICS Microbial Community DNA Standard II (Zymo Research)) were used as template in a two-step PCR amplification described in the application note, with 35 cycles of amplification for the first-round PCR and a further 8 cycles with Nextera XT index primers set A and B (Illumina, San Diego, CA, USA) to allow pooling all samples in a single sequencing run. Reactions were cleaned with AMPure XP beads, before each was run on a Tapestation 4200 (Agilent Technologies, Santa Clara, CA, USA) using DNA-1000 reagents (Agilent Technologies). Samples were pooled based on concentrations measured on the Tapestation, before a final cleanup on 1% agarose gel to remove residual primer dimer artifacts, with recovery of DNA using QIAquick gel extraction columns (Qiagen, Hilden, Germany). Sequencing was performed on a MiSeq (Illumina) with 300 bp paired end reads (v3 reagents), with PhiX library (Illumina) blended to 30% and cluster density reduced to 80% recommended levels, running RTA v1.18.54 and bcl2fastq v2.21.0.422. A total of 18,642,611 read pairs passing filters were generated, and demultiplexed to yield an average of 116,511 read pairs per sample (range 41,922 − 377,276).

### 16S rRNA sequencing data analysis

The demultiplexed paired-end raw reads from a total of 131 samples (110 samples and 21 controls) were analyzed using the DADA2 R package^47^. Primers and adapter sequences were trimmed, and reads were filtered to remove the low-quality tails at the truncation length of 250 bp for both forward and reverse reads, using the parameters of maxEE of 2 and truncQ of 2. The quality-filtered reads were dereplicated and denoised using the core DADA2 algorithm and the paired reads were merged. An amplicon sequence variant (ASV) table was constructed, and chimeric ASVs were identified and removed. Taxonomy assignment to the ASV table was performed using the default settings of the Ribosomal Database Project (RDP) Naive Bayesian Classifier algorithm^48^ implemented in DADA2 R package with Silva prokaryotic SSU taxonomic training data formatted for DADA2 with species (release-138.1)^49^.

### Decontamination of the sequences

A Phyloseq object was created from the ASV, taxonomy, and metadata tables using the phyloseq R package^50^. Taxonomy-based filtering was performed to remove the features that were assigned to Archaea, Chloroplast, and Mitochondria, and only the ASVs assigned to at least the class level were kept for further analysis to avoid the host sequence contamination. Contaminant sequences were identified and filtered out based on the prevalence method using the decontam R package^51^. All sequences that were more prevalent in negative controls than in positive samples were identified as contaminants (using the threshold of 0.5). The mock community sample dilutions were used to evaluate the unexpected sequences across the dilution series during the decontamination process. The decontaminated ASV table was further filtered to keep the ASVs that are present in at least 5% of the total samples.

Alpha diversity indices of species richness (observed features and abundance-based coverage estimators) as well as species richness and evenness (Shannon diversity index)^52^ were estimated using the estimate_richness function in the phyloseq R package and plotted using the ggplot2 R package^53^. The pairwise comparisons were done using the Wilcoxon rank sum test with continuity correction, and the Holm correction was used as the *p-value* adjustment method.

### Unsupervised and supervised analysis of the microbiome

The filtered ASV table, which was agglomerated at the genus level, was pre-processed using the multivariate statistical framework mixMC^54^. Basically, one is added to all the values to deal with zeros, and ASVs with low counts were further filtered out using a cut-off of 0.01%. The Centered-Log Ratio (CLR) transformation was performed. The datasets were processed using the mixOmics R package^55^ to explore the effect of ewe breed, as well as estrous phase, on the cervical microbiome. An unsupervised analysis (PCA), after removal of individuals with < 500 reads, was used for dimensionality reduction and data exploration. A supervised analysis, sparse partial least squares-discriminant analysis (sPLS-DA)^56^, was used to select the most discriminative genera to classify the samples from different breeds sampled at a natural or synchronized estrus at the follicular phase.

## Electronic supplementary material

Below is the link to the electronic supplementary material.

Supplementary Material 1

Supplementary Material 2

Supplementary Material 3

Supplementary Material 4

Supplementary Material 5

Supplementary Material 6

## Data Availability

All data generated or analyzed during this study are included in this published article and its supplementary files. The sequence data were deposited in the Sequence Read Archive (SRA at https://www.ncbi.nlm.nih.gov/) under Bioproject Accession Number PRJNA1128062 (https://www.ncbi.nlm.nih.gov/bioproject/PRJNA1128062).
