# Supplementary material for: The cervical microbiome of ewe breeds with known divergent fertility following artificial insemination with frozen-thawed semen
Source: Sci Rep. 2025 Apr 26;15:14614. doi: 10.1038/s41598-025-97735-4 (PMC12033225; doi:10.1038/s41598-025-97735-4)

Supplementary Figures 1-5

The cervical microbiome of ewe breeds with known divergent fertility following artificial insemination with frozen thawed semen

Simen Foyn Nørstebø[1†], Sabrina Rodriguez-Campos[1†*], Özgün C. O. Umu[1], Laura Abril Parreño[2,3], Marianne Dalland[4], Gregor Duncan Gilfillan[4], Sean Fair[5], Anette Krogenæs[6]

[1] Bacteriology and Mycology Unit, Department of Paraclinical Sciences, Faculty of Veterinary Medicine, Norwegian University of Life Sciences, Elizabeth Stephansens vei 15, 1433 Ås, Norway.

[2] Physiology of Reproduction Group, Department of Physiology, Faculty of Veterinary Medicine, International Excellence Campus for Higher Education and Research (Campus Mare Nostrum), University of Murcia, Murcia, Spain.

[3] Institute for Biomedical Research of Murcia, IMIB-Pascual Parrilla, Murcia, Spain.

[4] Department of Medical Genetics, Oslo University Hospital and University of Oslo, Oslo, Norway.

[5] Laboratory of Animal Reproduction, Department of Biological Sciences, Biomaterials Research Cluster, Bernal Institute, Faculty of Science and Engineering, University of Limerick, Limerick, Ireland.

[6] Reproduction Unit, Faculty of Veterinary Medicine, Norwegian University of Life Sciences, Ås, Norway.

[†]These authors contributed equally to this work and share first authorship.

*Corresponding author: S. Rodriguez-Campos, sabrina.rodriguez@nmbu.no

Supplementary figures

Supplementary figure 1. Median cervical bacterial abundances of Suffolk, Belclare, Fur, and Norwegian White Sheep (NWS) at the follicular (left) and luteal (center) phases of a hormonally synchronized estrus, and at the follicular phase of a natural estrus (right). Data are presented as log10 16S rRNA gene copies per sample with the dashed line indicating the median bacterial abundance levels in the blank extraction controls. [ab]Different superscripts differ significantly between ewe breeds within each phase of the cycle (P<0.05).

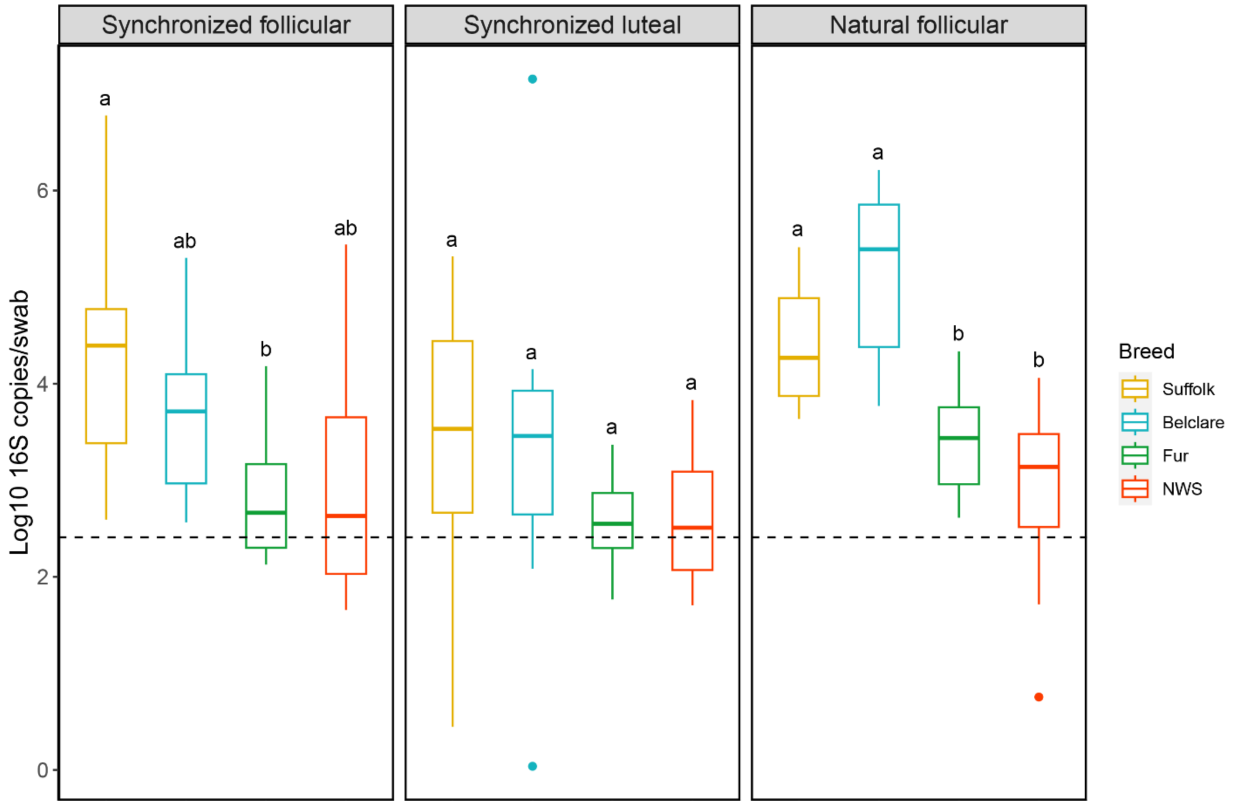

Supplementary figure 2. Bar plot showing reads classified as identified contaminant amplicon sequence variants (ASVs); ASVs filtered due to identification as Archaea, chloroplasts, mitochondria, or due to presence in less than 5% of the total number of cervical samples; and ASVs representing the mock community in the control samples.

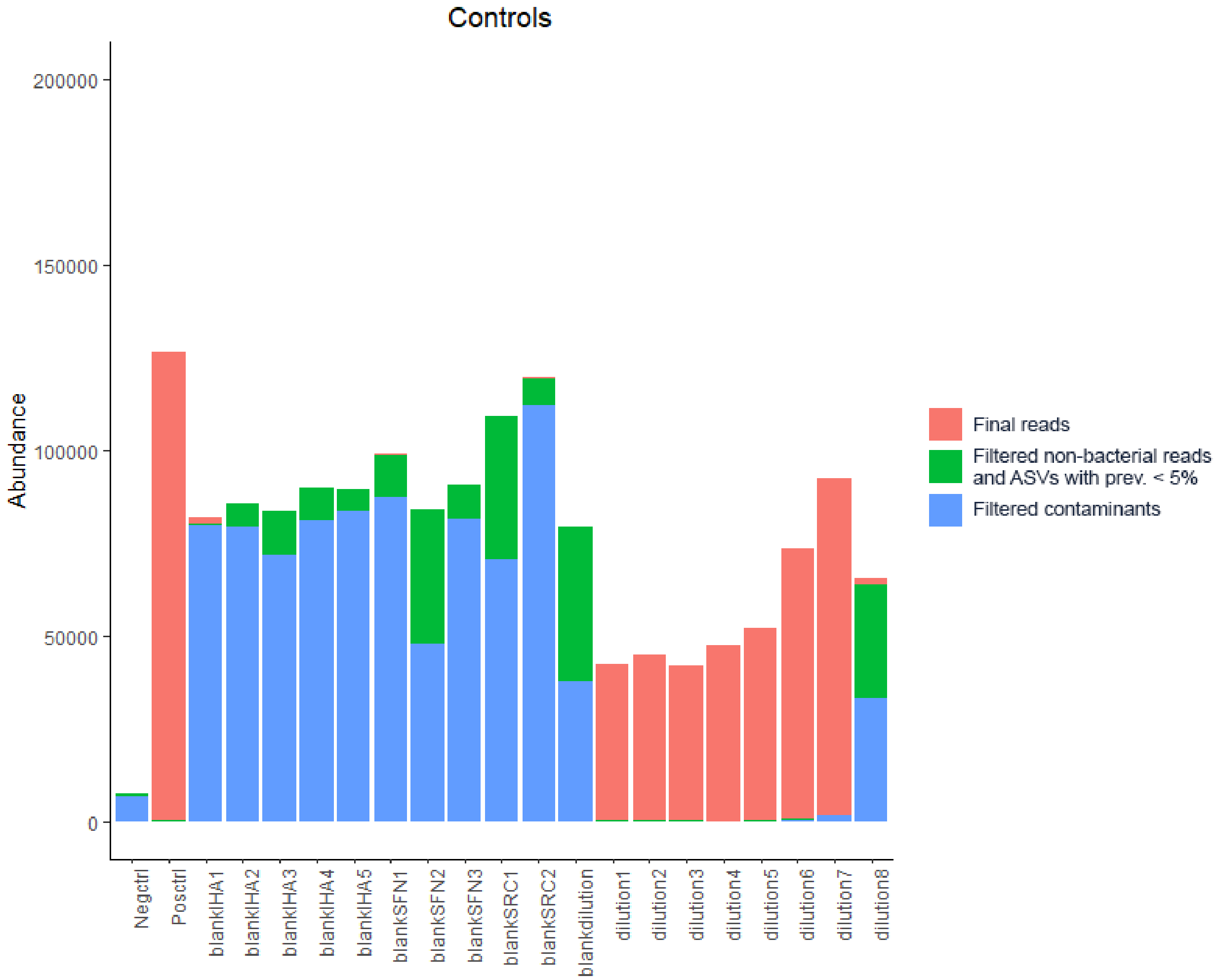

Supplementary Figure 3. Visual comparison by supervised partial least-squares discriminant analysis (sPLS-DA) of the cervical bacterial microbiome in Suffolk (n = 18), Belclare (n = 19), Fur (n = 19) and Norwegian White Sheep (NWS; n = 20) at the follicular phase of both a natural estrus and a hormonally synchronized estrus.

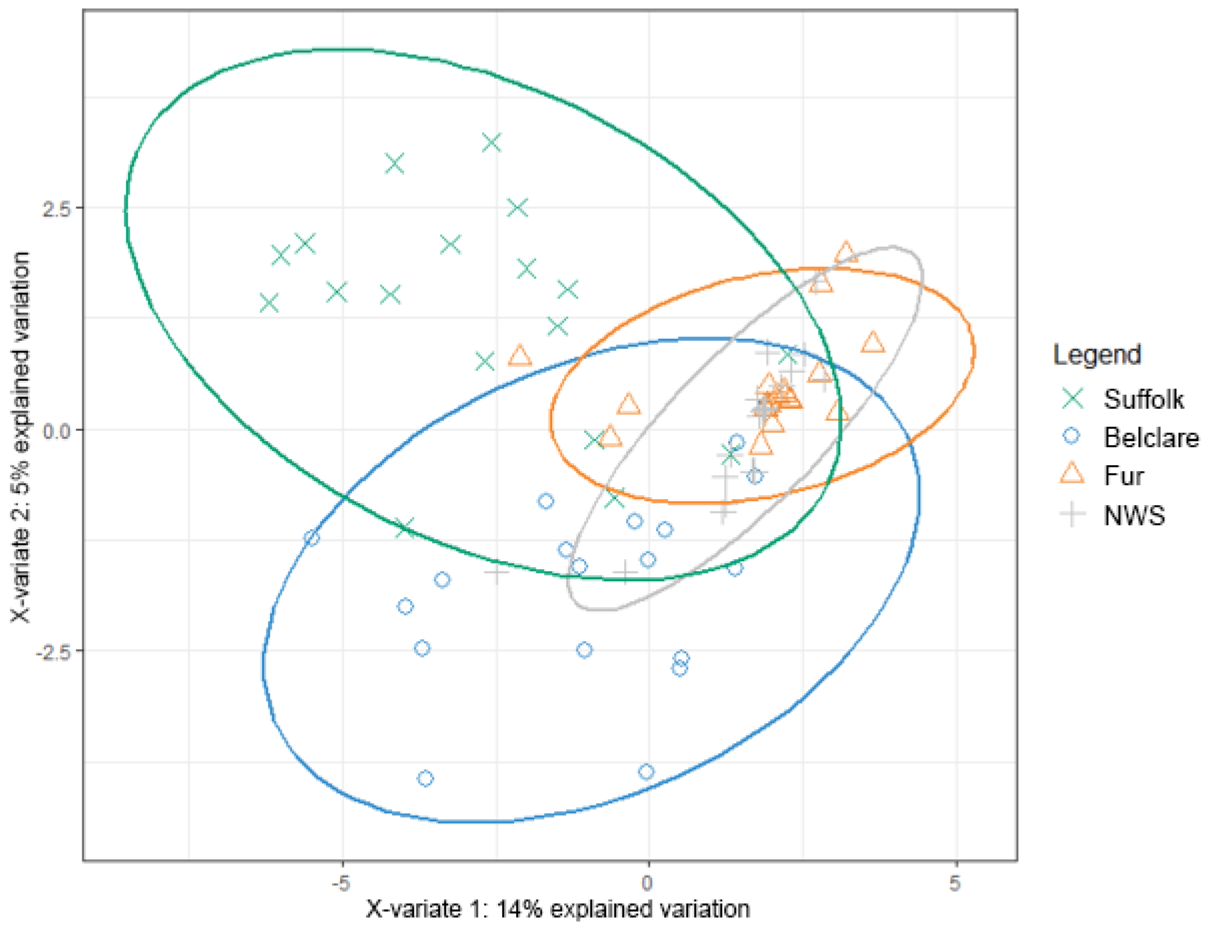

Supplementary Figure 4. Visual comparison by supervised partial least-squares discriminant analysis (sPLS-DA) of the cervical bacterial microbiome in different breeds sorted by the follicular phase of either a natural or a synchronized estrus in Suffolk (natural: n = 8; synchronized: n = 10), Belclare (natural: n = 9; synchronized: n = 9), Fur (natural: n = 10; synchronized: n = 9), and Norwegian White Sheep (NWS; natural: n = 10; synchronized: n = 10).

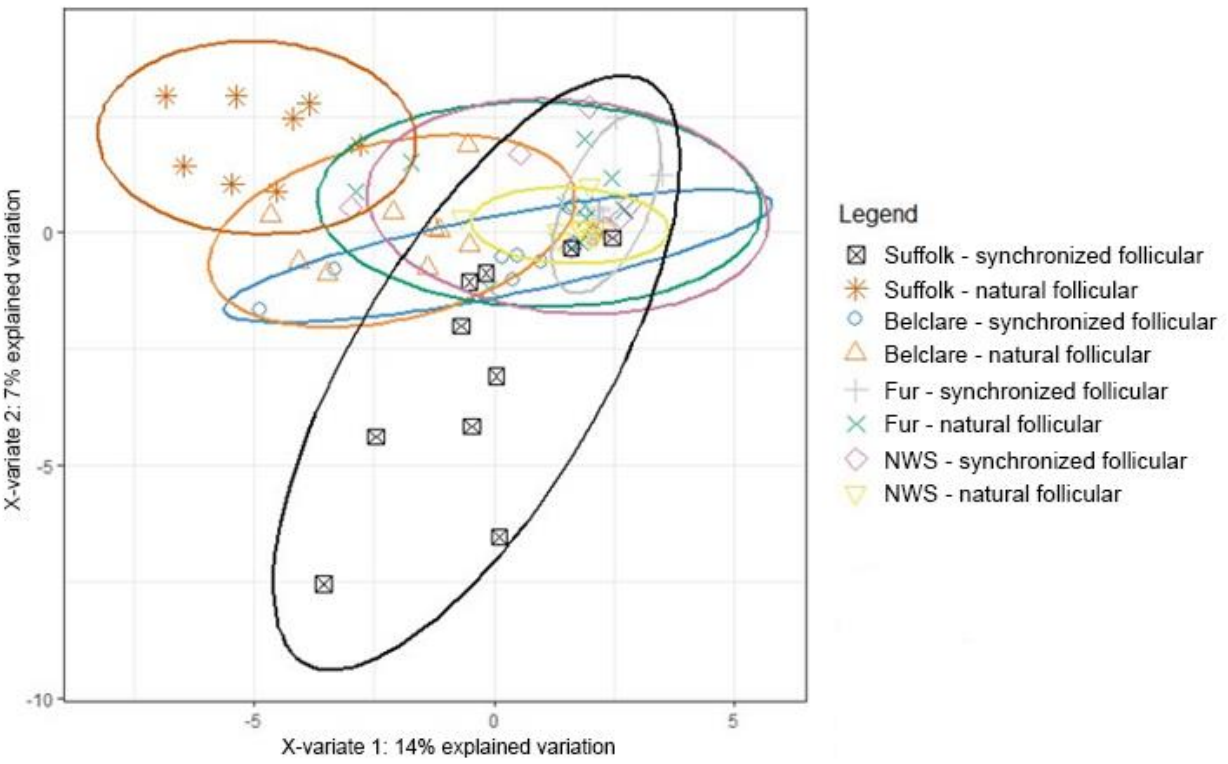

Supplementary Figure 5. Linear discriminant analysis effect size (LEfSe)-like plot generated using mixOmics. The plot illustrates the contribution to the first component of the supervised partial least-squares discriminant analysis (sPLS-DA, SupplementaryAdditional Figure 4) for ewes sampled at the follicular phase of either natural (Nat_Fol) or synchronized (Ind_Fol) estrus, as presented in Supplementary Additional Figure 4. Suffolk (natural: n = 8; synchronized: n = 10), Belclare (natural: n = 9; synchronized: n = 9), Fur (natural: n = 10; synchronized: n = 9), and Norwegian White Sheep (NWS; natural: n = 10; synchronized: n = 10).

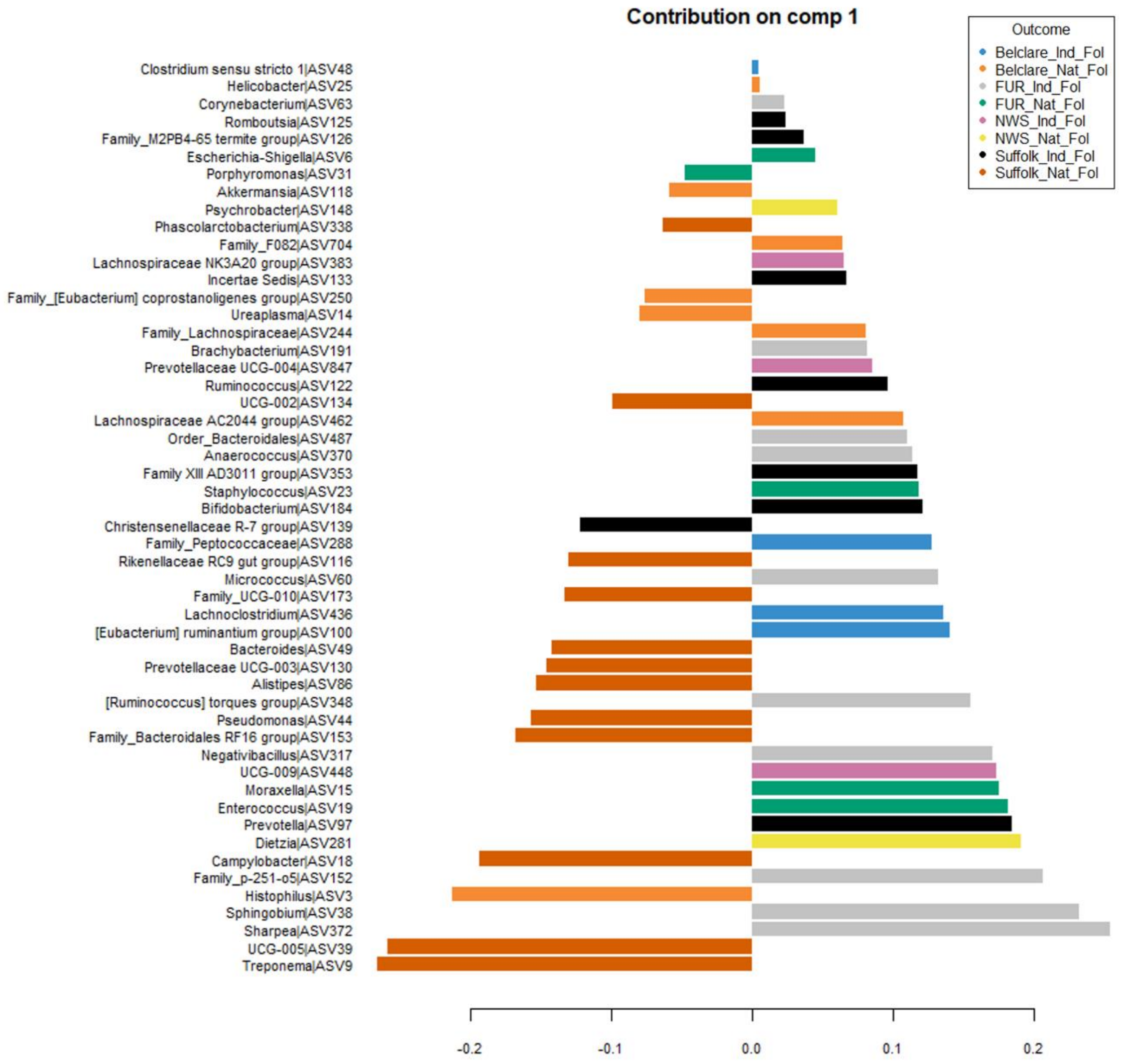

Supplement: Supplementary file 1 — Supplementary Material 1 [file 41598_2025_97735_MOESM1_ESM.pdf]
